# Notes on Myotomy and Tenotomy

**Published:** 1841-10-30

**Authors:** Reynell Coates


### NOTES ON MYOTOMY AND TENOTOMY.

BY REYNELL COATES, M. D.

To the Editors of the Medical Examiner.

Gentlemen,—My last communication concluded with an attempt to answer the objection so often urged against simple mechanical extension in club-foot, namely, its tendency to produce spasmodic resistance, or, in other words, an increase of the spasmodic action of the muscles already existing.

I pointed out the fact that, according to the usual course of events, the muscles become affected with marasmus from inactivity, until, after a certain length of time, their structure is so far altered as to render them incapable of troublesome spasm. The resistance to the extension is then no longer the consequence of the *action* of those organs, but their positive *shortening* by a change in their nutrition analagous to that which leads to the occlusion or coarctation of fistulous and other false passages, the abdominal rings in hernia when radically cured, &c.* But parts reduced to a condition nearly resembling simple, though condensed cellular tissue, yield readily, and grow rapidly under the action of moderate extending forces, and hence—so far as spasm is concerned—the propriety of the treatment of club-foot by properly regulated machinery alone depends solely on a question of time.

But, in certain cases, the contracted muscles undergo a series of changes very different in their effects from mere local marasmus:—the cellular tissue of these organs, instead of remaining the seat of a diminished, though altered deposit of fleshy fibre, becomes loaded with condroid matter analogous to that which constitutes the bones in the second stage of their developement. I have seen this transformation rendered sufficiently obvious in two cases of varus. Now, the stretching of a large mass of pseudo-cartilage is a very different affair from the elongation of a mere wasted muscle; and, here the operation of tenotomy is plainly indicated; not that the mechanical process is perfectly hopeless, but because the time lost in the former attempt would far outweigh the dangers and the ultimate weakness resulting from the after consequences of so trifling an operation as tenotomy.

It is folly to pretend that, even when there exists spasms of the extensors, their tendon can be divided without serious diminu-

* I have elsewhere argued at length against the doctrine of the radical cure of hernia by adhesive inflammation. See Final Report on the Radical Cure of Hernia, by a Committee of the Philadelphia Medical Society,—*American Journal of the Medical Sciences*, Aug. 1837, also Letter to Heber Chase, M. D., in *Chase on Hernia*, page 107.

tion of power for ever after: but when the chondroid degeneration just described has taken place, the evil is reduced to its minimum; because time is then allowed for the union of the extremities of the divided tendon to become firm and fibrous before tonicity of muscle can be restored, and only two or three inches of the new connecting medium intervenes, even when the foot is brought down immediately. When there is simple marasmus of the parts, on the contrary, it has been stated in the last note that some functional power usually remains; and under such circumstances, the muscular fibres must continue gradually to retract the superior portion of the tendon, from the moment of the operation up to the time when the new bond of union is rendered fibrous—a period of time measured at least by weeks, if not by months. Most of the writers on tenotomy have neglected this danger entirely; and as I must necessarily be somewhat more diffuse than usual at this point in the progress of our argument, I will venture to introduce a few illustrations of its importance.

A young class mate, who is still within the range of my personal acquaintance, had the misfortune to meet with a severe fall upon his back, when about thirteen years of age. The point of one elbow struck the hard ground violently in consequence of his efforts to save himself. The arm hung useless, and the elbow was much swelled. A practitioner, unused to surgical cases,—having been resident during all his professional life in the country, and never having been duly attentive to any hospital clinic—examined the part, pronounced the case a sprain, and ordered the arm to be suspended in a sling and regularly bathed!—The olecranon was broken, and drawn up some considerable distance! At the end of five or six weeks, according to the young man's account, the nature of the injury was discovered; but this was too late for remedy. The forearm could then be flexed with freedom, but could not be perfectly extended except when some other individual assisted the patient. Some months after the reunion of the fragments, the lad met with another fall, and in his involuntary efforts to avoid the consequences with his uninjured and more useful limb, the other olecranon struck the ground and was fractured.

The partner of his former physician attended the case, committed the same error of diagnosis, adopted the same mode of treatment, and effected nearly the same result!

This young gentleman is now nearly forty years of age. The superior fragments of both ulnæ are separated from the inferior fragments,—one to the distance of nearly an inch, and the other nearly two inches. The former, though connected with the inferior fragment by a well defined cellular band, is firmly adherent to the back of the os humeri, and the corresponding triceps muscle is scarcely distinguishable, even as a rudiment,—the other moves with perfect freedom, and the corresponding triceps, though disproportionately small, is as tense and active as its bulk would lead us to expect. Yet both forearms are maintained habitually in the semi-flexed attitude of sleep, in consequence of the unbalanced tonicity of the triceps and brachialis internus muscles. Both arms can be flexed voluntarily to their full extent, but that in which the fragment is adherent, is dependent almost exclusively on the force of gravity for its power of extension. Even on the side on which the triceps continues active, the limb cannot be fully extended, because the forearm being retained almost constantly in a flexed position, the posterior part of the elbow joint appears to have been considerably modified in form for want of functional exercise.

Again—An intelligent patient was admitted into the Pennsylvania Hospital, some years before my former connection with that institution commenced, (1819) with fracture of the patella. According to his own statement, the fragments were separated less than half an inch at the time of his discharge, and the limb was firm and useful. He left the house contrary to the advise of his surgeon, before the latter felt perfectly sure of the strength of the new deposits.

At the time of the great northern progress of President Jackson, this man lost an arm by the wad or the bursting of a piece of ordnance in firing a salute. He was again admitted into the Hospital in order to undergo an amputation, and I enjoyed the opportunity of examining the knee. The fragments were then separated to the extent of five and a half inches! The connecting band was flat where it was joined to the fragments, but took the form of a tense ligament, nearly round, and about as thick as the tendo Achilles, as it became gradually attenuated towards the middle of the interval between them. The limb was still useful, probably in part owing to remaining connections of some fibres of the two vasti with the lower fragment of the patella and with the fascia of the leg; but the rectus itself was apparently undiminished in lateral diameter, though shortened to the extent of more than one-third the entire length of its fleshy fibres! He was able to walk without limping or betraying lameness, but he expressly stated that he had been subjected to many falls, was compelled to exercise great caution in descending, and felt great fatigue and difficulty in ascending flights of stairs, in consequence of the feeble power of the extensors of the leg.

It is hardly necessary to prove the position that human nature strongly inclines us all to place full faith in the truth of what we evidently desire should be true. All have felt this disposition, and it is one of the most important, as well as difficult tasks in professional self-government to restrain it effectually—

but it is singular that some of those most ardent in the praise of almost indiscriminate tenotomy should be induced to act with such extreme incaution as they have done in drawing conclusions favourable to their views, in the very teeth of obvious general laws. There are those who, in arguing in favour of "bringing the heel down" immediately after the division of the tendon, venture to assert in broad terms,—acting evidently upon some vague generality founded on *the occasional presence of spasm in recent club-foot*—that although the muscle contracts instantly and forcibly at the moment of division, it soon relaxes itself again, *and suffers the divided extremities to approach each other until they come nearly or completely into contact.*—As if "step-dame nature," in her motherly affection for the most unfortunate and deformed among her offspring, had kindly uttered an especial order in council, modifying her Medean and Persian edicts, in amiable condescension to the intreaties of her favourites, the tenotomists! What pity that she has been less gracious to those who meet with fractures and luxations!—that we cannot persuade the muscles politely to relax and render tolerable and useful *one thousand and one* most ingenious but now useless contrivances for fractures of the thigh, and of the patella or olecranon, to say nothing of the facilities which such generosity would yield us in the removal of tumours and operations on the parietes of the abdomen, by allowing us fearlessly to cut and turn aside either muscles or tendons when they stand in our way,—well assured that if edges of the skin be brought rightly into coaptation, these *more tractable* organs would speedily "rattle into ranks," and throw no difficulties in the way of *le reunion immediate!*

I have said that, even when a muscle has exchanged the usual and appropriate deposit of fleshy fibre for something analagous to that formation which is observed between the apophyses and the bodies of the bones of young children, the whole skeleton of the condrilomatous fishes, parts of that of certain reptilia, &c.—the restitution of the foot to its normal position is still far from hopeless, when treated mechanically, though such treatment is decidedly contra-indicated in consequence of the extreme tediousness of its operation under such circumstances. If disposed to be governed by high authority in the face of facts of daily and hourly occurrence, it would be easy for an antagonist to smile at this position; for some high authorities have taught the doctrine that a tissue once structurally altered in character cannot resume its former condition, though no case of considerably misplaced fracture ever recovers without producing precisely this order of structural alterations in surrounding parts. As strong examples, of a similar nature, it may be well to mention two cases, the first of which was very remarkable.

A journeyman shoemaker, then resident in this city, applied to me some years ago, for advice on the condition of his arm and elbow-joint. The anterior portion of the right arm, from the neighbourhood of the wrist to that of the elbow, was greatly swollen and very hard. The hand and wrist-joint had lost all power of voluntary flexion, though the fingers, which were placed habitually in the demiflexed position, could be extended almost perfectly by a strong effort. The posterior portion of the forearm was nearly in a natural condition. On the anterior part were four round orifices, widely distant from each other, and resembling those seen usually in necrosis, and a fifth, of similar character, was seen about an inch *above the external condyle of the os humeris.*

The patient stated that the disease had been gradually approaching its then existing condition for about four months. An examination was made under the idea that there existed simultaneous necrosis of the radius and ulna. The probe entered but a short distance perpendicularly into each of the orifices before it took a deep and devious course somewhat in the general direction of one or other of the distant openings. In its passage, it obviously penetrated a canal through a substance as firm as cartilage, although, in several places, it evidently crossed the usual track of various muscles of the forearm, and occasionally grated, or rather jarred upon spicula of bony matter, which no where communicated precisely the sensation of a contact between silver and denuded bone. Confirmed in the erroneous diagnosis that the case was one of necrosis in which the sequestra could not be felt, on account of the tortuosity of the fistulæ and the enormous developement of the periosteum, the general health of the patient was carefully attended to, and the disease was treated solely by the adoption of constitutional measures for more than three months. But the impatience of the man, and his urgency for an operation was such as to induce a more rigorous examination, and finding a communication between two of the orifices on the radial side of the arm, one near the bend of the elbow, and the other about three inches lower down, I laid this track entirely open by means of a long director and the probe-pointed bistoury, which latter met with very considerable resistance from the mass of chondroid matter, nearly an inch in depth, through which it passed. This incision was made in the hope of finding a route by which the condition of the radius might be ascertained—but, no where was the probe permitted to approach the bone. About the centre of the track, another canal branched off towards a third external orifice on the ulnar side of the forearm.

A critical examination of the incision displayed a structure in which cartilage or chondroid matter predominated, intermixed with scattered signs of altered muscular fibre, in the condition seen in marasmus from inaction, and numerous spicula of bone. It was now evident that the case was not of the character of necrosis; and, as it seemed hopeless to expect union by the first intention, poultices were ordered, and the constitutional treatment continued—the administration of the mineral acids and a rather generous diet being its principal features.

Much to my surprise, the bottom of the incision became rapidly filled with healthy granulations. In a few days the whole forearm seemed to be slightly softer to the touch, and there was an increased yielding to compression in the immediate neighbourhood of the wound. Encouraged by this circumstance, I traced and laid open the sinuous canals, throwing all the orifices into one, at the expense of seven or eight inches of incision, laying nearly bare at one point the external lateral ligament of the elbow, but no where meeting with well formed muscular fibre, or finding any reason to infer disease of the bones or periosteum. Pressure by the roller, and poultices over the roller, were employed for a considerable time with the double intention of maintaining suppuration and promoting absorption; until, finding the hardness rapidly disappearing from the forearm, the dressings very troublesome, and the ununited remains of the incisions were healed under the action of adhesive strips, and dependence was placed solely on the roller. In a few months the flexors and pronators of the forearm *recovered their functions to a very considerable extent.* No doubts remained of the reconstruction of the proper tissues, the case approximated to a perfect cure, and the patient, probably dreading his bill, very wisely *ran away!* I must apologize for the want of more accurate specification of dates in this and other narratives of cases, by stating that the proper notes, together with those of numerous other highly interesting occurrences, and the manuscripts of three years of labour spent upon a surgical bibliotheca, appear to have been lost in the confusion growing out of the irregular movements of the Navy Department in relation to the original South Sea Expedition.*

The second case, to which allusion has been made, was one of torticollis, occurring in the practice of my friend Dr. Barnes, of Poughkeepsie, N. Y., who has several times performed the operation of myotomy in that species of deformity. In this particular instance he informed me that the contraction of the sterno-cleido-mastoideus muscle was so great that the mastoid process was drawn to considerably within two inches of the sternum, (the nearest practicable voluntary approximation, I find by measurement, to be from three inches to three inches and a half.) On dividing the muscle, it was found to be entirely transformed into chondroid matter. The head was gradually restored nearly to its natural attitude by mechanical means, which slowly overcame the established malposition of the cervical vertebræ consequent upon the long continued twisting of the neck, and the case was still under treatment at the time of our conversation (September, 1840.) The muscle had been then regenerated so far as to give very obvious proof of its functional action.

Having now pointed out two cases of exception to our argument in chief,—the discussion of the relative advantages and disadvantages of tenotomy in the mechanical treatment of club-foot,—one of which cases is the actual existence of spasmodic action equally interdicting both methods of treatment, and the other consists in a chondroid transformation of the contracted muscles, a very rare affection in which tenotomy is decidedly indicated, we have arrived at a point which requires me to close for the present.

* It will require all the talent and industry of the few able naturalists attached to the present Expedition to repay the science and the arts of the country, for the time, money, life, and material sacrificed to the folly of those who crushed the former undertaking.

